# The History and Capabilities of Herbal Simples

**Published:** 1889-12-28

**Authors:** W. T. Fernie


### The History and Capabilities of Herbal Simples.

By W. T. Fernie, M.D.

### I.—INTRODUCTORY AND HISTORICAL.

Virgil tells us that Apollo, the God of Medicine, once offered to endow Iaspes, his favourite pupil, with his own high attributes of augury, music, and skill in archery. But Iaspes preferred to acquire a knowledge of simple herbs and of their curative uses in sickness. By possessing this knowledge he afterwards saved the life of Æneas when grievously wounded by an arrow, and he averted death from that hero by using the plant dittany, smooth of leaf and purple of blossom, which he plucked on Mount Ida.

The mantle of Iaspes subsequently fell on our own British forefathers, with whom, from early times, native medicaments found always much favour and esteem. To any such remedy, consisting of one ingredient only, and that for the most part a herb, they gave the name of a simple. The Anglo-Saxon verb, "drigan," to dry, when applied to herbs, originated our modern word "drug," and the medicines of our ancestors were chiefly dried herbs. The primitive medicinal pill was the hog-louse, or millepede, found in dry gardens, which rolls itself up into a ball when touched. From three to twelve of these were given in Rhenish wine, for a hundred days together, to cure all sorts of cancers. Such was the reverent simplicity of ancienter times. A book of simples was published by Dr. Bulleyne, in 1568, and formed part of his bulwark of defense against all sickness, sornes, and woundes that dooe daily assaulte mankinde. Again, in 1652, Nicholas Culpeper, student of physick, issued a compleat method whereby a man may cure himself, being sick, for three pence charge, with such things only as grow in England, they being most fit for English bodies. Also, in 1656, the art of simpling was produced by William Cole, author of "Adam in Eden; or, Nature's Paradise," and this exclusively advocated the employment of our native herbal medicines. In Shakespeare's time a street, still named Bucklersbury, in the City of London, was famous for its number of druggists who sold simples and sweet-smelling herbs. So Sir John Falstaff, in "The Merry Wives of Windsor," flouts the effeminate fops of his day as lisping hawthorn-buds that smell like Bucklersbury in simpling-time. But of all English herbals wherein plants have been discussed as native remedies, by far the most popular was the large work which Gerard published during Elizabeth's reign in 1597. Its quaint, engaging style, and its queer astrological notions, together with its admirable woodcuts of the plants described, have made this book a standing favourite with the English community ever since. Gerard had a large physic garden near his house in Old Bourne, Holborn, and there is in the British Museum a letter drawn up by his hand asking Lord Burghley to advise the university of Cambridge to establish there a physic garden for encouraging the faculties of simpling. Further evidence shows that the same affection for simple herbal medicine holds good equally to-day amongst ourselves, specially with the humbler classes. This fact may be verified on any Saturday night in one or another more crowded commercial thoroughfare, whether in London or in a big city like Manchester, where hucksters congregate thickly, with their handbarrows of shell-fish, vegetables, and other miscellaneous perishable commodities. Here the herb doctor, under a flaring lamp of smoky petroleum, and amid an imposing show of bottles, pots, roots, and dried plants, pitches the patter, more or less glibly, as he may have the gift of the gab, and finds a ready sale for his herbal drink, his hop bitters, his vegetable pills, and his botanical powders, with the rest of his quack nostrums. Honest, simple-minded working-men and credulous youths purchase these various concoctions with blind faith, and swallow them with astonishing avidity. Lately, at one of these stalls, in the Edgware-road, on a fine Saturday night, between the hours of seven and eleven o'clock, I calculated that, at the rate of sale which took place under my own observation for an hour, at least some four or five pounds must have been taken altogether.

Concerning the herbal simples of former times, it must be specially noted that solely from hearsay, experience, and observation—and not on the basis of any scientific investigation or research—did they become credited with the curative virtues they were supposed to possess. It has remained for the modern chemist, after some centuries of steady medical progress, to analyse and reveal the true nature and foundation of the healing qualities really exercised by these simples. Let me take, for example, our common English watercress, of which the botanical name is nasturtium, from "*nasus tortus*," a nose set awry, by the pungent juices of the plant. From very early times this herb has enjoyed a popular reputation for being good against the scurvy, for opening obstructions of the glands, and for purifying the humours of the body. Gerard, in 1597, says, "The watercresse, being boiled in wine, or milke, and drunke for certaine daies together, is very good against the scurvy, or scorbute." And in the following century the juice of the watercress was prescribed, with the juices of scurvygrass and Seville oranges, as a popular remedy for scurvy under the name of spring juices. But not until recently have chemists shown us the curative presence in the watercress of sulphur, phosphorus, iodine, and iron as constituent principles, which are actual antidotes to scrofula. Likewise, John Wesley, in his "Primitive Physic; an Easy and Natural Method for Curing most Diseases," 1769, advised boiled carrots for asthma, a decoction of boxwood for baldness, the juice of nettles for blood-spitting, clivers, or goosegrass, for an open cancer, and six middling cobwebs for the cure of ague. In his day nothing but tradition, practice, and shrewd observation led him to extol these native remedies. It is only of late that chemists have found that the carrot, when wild, possesses a volatile principle which stimulates the bronchial membranes, and promotes expectoration; that the boxtree furnishes buxine, which specially excites the nerves of the hair bulbs; that the nettle is endowed as to its stinging hairs with formic acid, which serves to arrest bleeding, that the goosegrass is rich in tannin for the relief of cancerous sores, and that the spider's web exercises a particular virtue against ague, because of its containing a medicinal substance analogous to and isomeric with quinine.

It is my purpose, with the consent of the editor of this journal, to reconsider, in a short series of articles, the domestic remedies which were most in vogue with our forefathers as herbal simples; and to show with what reasonable prospect of success their curative employment may be resumed or retained in the present day, under the trustworthy guidance of recent chemical analysis, and of precise scientific provings. I shall discuss the simples almost alphabetically whilst naming the genus and natural order to which each medical plant under consideration belongs, but shall pass over the highly poisonous plants, such as the foxglove, deadly nightshade, hemlock, henbane, monkshood, and the like, these being beyond the domain of herbal simples, and only to be used under the advice and care of a qualified prescriber. But no similar scruples operated formerly to deter ignorant pretenders from supplying dangerous drugs to the public; as, for example, in the reign of Queen Anne, when a shoemaker, named Trigg, left off mending soles, and took to the curing of bodies. Unfortunately, he soon caused the death of several persons, and was transported for this crime. Also, in the same reign, one, William Forrester, was severely punished for selling the bitter apple, colocynth, to cure scurvy; and one, Simon Foreman, for administering the wild cucumber, to cure dropsy. Addison, in the *Tatler*, tells of a doctor, in his day, who had arrived at the knowledge of the green and red dragon, and had discovered the female fern-seed. About the same date, elephant's milk was imported expressly from the deserts of Africa by one, Doctor Campbell, as a panacea surpassing all other remedies. Let the greatest invalid drink of this; and he shall live! Likewise, the reanimating solar tincture, or pabulum of life, was invented by Dr. Sibley, each dose of which had to be elaborated from the four elements throughout many weeks and which was an infallible cure for all the bodily ills to which our flesh is heir. But, alas for their credit, each of these wonderful nostrums was subjected to analysis at the instance of the *Gazette of Health*. The elephant's milk was found to be composed of gum mastic, dissolved in spirit of wine, which, of course, became milky in appearance when mixed with water. And the solar tincture proved to be highly rectified spirit of wine, flavoured with the essential oils of orange and of the sweet flag root.

